# Assessing the impact of the five senses on quality of life in mucopolysaccharidoses

**DOI:** 10.1186/s13023-020-01368-x

**Published:** 2020-04-19

**Authors:** Roberto Giugliani, Paul Harmatz, Shuan-Pei Lin, Maurizio Scarpa

**Affiliations:** 1 Department of Genetics/UFRGS, Medical Genetics Service/HCPA, DR BRASIL Research Group/HCPA, and INAGEMP, Rua Ramiro Barcelos 2350, Porto Alegre, RS 90035-903 Brazil; 2 Department of Gastroenterology, UCSF Benioff Children’s Hospital Oakland, Oakland, CA USA; 3 Department of Genetics and Metabolism, MacKay Children’s Hospital, Taipei, Taiwan; 4 Regional Center for Rare Diseases, University Hospital of Udine, Udine, Italy

**Keywords:** Hearing, Mucopolysaccharidosis, Patient-reported outcomes, Quality of life, Review, Senses, Smell, Taste, Touch, Vision

## Abstract

**Background:**

The mucopolysaccharidoses (MPSs) are lysosomal storage disorders associated with progressive multi-organ and skeletal abnormalities. Clinical manifestations can affect each of the five senses: hearing, vision, smell, taste, and touch.

**Main body of the abstract:**

On 24–26 May 2018, 46 specialists with expertise in managing symptoms of MPS and experts specialized in evaluating and managing impairments in each one of the five senses gathered in Lisbon, Portugal at the “MPS & the five senses” meeting to discuss how loss of one or multiple senses can affect activities of daily living (ADL) and quality of life (QoL) in MPS patients and best practices in evaluating and managing the loss of senses in these individuals. The meeting confirmed that MPS can affect the senses considerably, but how these impairments affect ADL and overall QoL from a patient’s perspective remains unclear. A better insight may be achieved by prospectively collecting patient-reported outcome (PRO) data internationally in a standardized way, using a standard battery of tools. To identify relevant PRO tools, a systematic literature review and a selection of existing published questionnaires, focused on adults with no intellectual delay, were performed after the meeting. The search strategy identified 33 PRO tools for hearing, 30 for speech, 125 for vision, 49 for touch (including pain and upper limb function), and 15 for smell/taste. A further selection was made based on several criteria, including applicability/relevance for MPS, applicability in different countries (languages)/cultures, availability in English, ease of use, validation, and normative data, resulting in a final set of 11 tools. In addition to these sense-specific PRO tools, a general QoL tool, the EuroQol (EQ)-5D-5 L, was selected to assess overall QoL and reveal coping behaviors.

**Short conclusion:**

MPS can affect each of the five senses, but current knowledge on the impact of sense impairments on QoL/ADL in MPS patients remains limited. Collection of data in a standardized fashion using sense-specific patient-reported outcome tools and a general QoL tool may fill the current knowledge gap.

## Background

The mucopolysaccharidoses (MPSs) comprise a group of lysosomal storage disorders caused by deficiencies in enzymes involved in the catabolism of glycosaminoglycans (GAGs) [1]. Patients with MPS exhibit an array of progressively worsening disease manifestations caused by GAG accumulation in tissues and organs throughout the body, including skeletal and joint abnormalities, cardiorespiratory disease, neurological disease, ocular abnormalities, and hearing loss [1]. Currently, there are 11 distinct subclasses of MPS disorders, each affecting a specific lysosomal hydrolase: MPS I (Hurler, Hurler-Scheie, and Scheie syndrome), MPS II (Hunter syndrome), MPS III (Sanfilippo syndrome, including subtypes MPS IIIA, IIIB, IIIC, and IIID), MPS IV (Morquio syndrome, including subtypes IVA and IVB), MPS VI (Maroteaux-Lamy syndrome), MPS VII (Sly syndrome), and MPS IX [1]. The GAGs that accumulate in these MPS subclasses differ considerably with respect to length, sulfation patterns, and other structural variations, leading to pathophysiological differences [2]. Clinical presentations and progression rates vary widely between and within MPS disorders.

The various clinical manifestations of MPS may considerably affect each of the five senses: hearing, vision, smell, taste, and touch [3–7]. However, little is known on how patients perceive impairments in the senses and how these affect activities of daily living (ADL) and quality of life (QoL). Patient-reported outcomes (PROs) that assess functioning (ADL) and QoL are becoming increasingly important in clinical practice and research to supplement clinical disease markers, and provide important insight into how patients perceive their condition and cope with their disabilities on a daily basis [8].

The “MPS & the five senses” expert meeting was held on 24–26 May 2018 in Lisbon, Portugal to discuss how loss of one or multiple senses can affect ADL and QoL in individuals with MPS, best approaches to evaluate loss of senses using PRO tools, and management of loss of senses in these individuals. A total of 46 specialists with expertise in managing symptoms of MPS and experts specialized in the evaluation and management of impairments in each one of the five senses attended this meeting; four adult patients with MPS testified about their impairments and the impact these impairments have on ADL and QoL.

The objectives of the present paper are to provide an overview of the overall findings of the “MPS & the five senses” meeting and to identify the most relevant PRO tools for assessing impairment in the senses and their impact on ADL/QoL in MPS patients.

## Impairments in the five senses in MPS

### Hearing and speech

Progressive hearing loss is a common feature of all MPS disorders, and is mainly due to recurrent otitis media, middle ear effusion, ossicle deformity, inner ear (cochlea) abnormalities and alterations in the auditory nerve [3, 9]. While young children with MPS generally present with conductive hearing loss, mixed hearing loss or pure sensorineural hearing loss tends to develop later in life as part of the natural history of MPS. Hearing impairment, but also oral manifestations (enlargement of the lips, tongue, and oral mucosa), abnormalities of the larynx and vocal tract, abnormal nerve function, rhinolalia, and/or cognitive issues can complicate speech, language, and communication [10, 11]. Untreated hearing loss may also lead to cognitive impairment [12]. Hearing and speech problems may affect social interactions and participation, development and learning, work, and overall QoL, and can have psychological consequences (e.g. depression) [13, 14]. Communicative disabilities can also considerably affect other people in the patient’s environment [14].

Because of the high prevalence of hearing impairment, regular monitoring for hearing loss in individuals with MPS is important. Hearing-specific questionnaires may be useful to assess the impact of hearing impairment on the patient’s QoL [13, 15]. Management of hearing loss in this patient population (e.g. ventilation tube insertion, hearing aids, cochlear implantation) depends on the type of hearing loss (conductive, sensorineural, or mixed), the degree of hearing loss, and age of onset. Ventilation tubes do not always normalize hearing in MPS patients with conductive hearing loss [16]; clinicians should not delay considering hearing aids for these patients. Reports about the effect of hearing aids and cochlear implants in patients with MPS are sparse [17]. Patients with speech problems may also benefit from ventilation tube insertion, amplification, and speech therapy [10].

### Vision

Individuals with MPS frequently present with ocular manifestations that can result in impaired vision and even blindness [4, 18]. Although ocular features have been described in all MPS disorders, they are particularly common in MPS I, VI, and VII [4]. Typical ocular manifestations of MPS include corneal clouding, astigmatism (mainly hyperopia), retinopathy, glaucoma, and optic nerve abnormalities (optic disc swelling, optic nerve atrophy), amblyopia, strabismus, and possibly cerebral visual impairment [4, 18]. Morphological changes in the eye generally develop very early in the disease course and are often already present at the time of diagnosis [18]. These alterations can be caused by excessive GAG storage in the cornea, trabecular meshwork, iris, ciliary body, retina, dura, sclera, optic nerve, extra-ocular muscles, and/or posterior visual pathway [4].

Visual impairment and blindness can considerably affect a patient’s independence, mobility, ADL, social interactions, education, work, and overall QoL [19–21]. Regular eye exams in MPS patients are essential to detect ocular abnormalities and allow proper management in an early stage [4, 18]. In addition, vision-specific PRO tools may provide information regarding potential impairments in vision and their impact on the patient [22]. Corneal transplantation (keratoplasty) showed good outcomes in MPS patients with corneal clouding, and should be discussed and recommended to these patients [23]. However, it should be noted that examination and surgical management of ocular manifestations of MPS can be challenging due to presence of masking concomitant symptoms (e.g. masking of visual field changes associated with glaucoma by superimposed visual field problems due to retinopathy), anesthetic risks, clinical progression, and social isolation [4, 18]. Patients with neurological decline or behavioral problems may be unable to cooperate in ophthalmological examinations [18].

### Smell and taste

There are several clinical manifestations of MPS that may cause impairments in smell and/or taste, i.e. adenoid hypertrophy, chronic rhinosinusitis, recurrent upper respiratory tract infections, thickened nasal cartilages, macroglossia, dental defects (such as dental caries, gingival inflammation, enamel hypoplasia, unerupted teeth, hyperplastic tooth follicle, anterior open bite, and condylar defects), and possibly neurodegenerative disease [5, 24–26]. In addition, continuous upper airway infection can cause chronic production of infected mucus, altering smell and taste. Tracheostomy, a procedure often performed in MPS patients with progressed upper airway disease, may give rise to impaired nasal function [27]. However, there is a lack of publications describing the impairments of smell and taste in patients with MPS.

Olfactory dysfunction may negatively affect one’s appetite, personal hygiene, social relationships, detection of hazardous odors (e.g. smoke, gas), and ADL (e.g. cooking), and may result in weight loss [28]. Therefore, it is important that clinicians are aware of the potential presence of impaired nasal function in MPS patients. Impairments in olfactory dysfunction can be identified using odor identification, discrimination, and threshold levels (e.g. using Sniffin’ Sticks) and PRO questionnaires [29]. Vaccinations, medical and surgical treatment of rhinosinusitis, and nasal saline irrigation may prevent or improve impairments in smell and taste in these patients [30–32]. Hyperosmolar nasal sprays should be used with caution, as experience with these sprays is mostly limited to healthy individuals. In patients with swollen mucosae, it may obstruct rather than free the nose [33]. It is important to be aware that adenoidectomy can be challenging due to difficult airway access in patients with limited neck extension, macroglossia and/or reduced mouth opening, and the risk of atlanto-axial subluxation [30].

### Touch (including upper extremity function and pain)

Musculoskeletal disease involving the upper limbs (i.e. skeletal and joint abnormalities), nerve compression syndromes (i.e. carpal tunnel syndrome [CTS], ulnar nerve entrapment [cubital tunnel syndrome], cervical cord compression), and central nervous system changes can result in impaired sensation and function of the upper extremities in MPS patients [6, 7, 34–36]. Skeletal and joint disease is particularly common in patients with MPS I, IV and VI, but also occurs in the other MPS disorders [35]. Typical skeletal findings in the upper extremities of MPS patients include bony and joint abnormalities in the fingers (claw hands, trigger finger) and forearms, and restricted joint motion in fingers, elbows, and shoulders [35, 37]. Wrist hypermobility is a typical and unique manifestation of MPS IVA, resulting in limited control of the wrist and weak grip strength [7, 38]. CTS is caused by compression of the median nerve in the carpal tunnel at the wrist, which is formed posteriorly by the carpal bones and anteriorly by the transverse carpal ligament [34, 38]. Apart from the median nerve, nine flexor tendons and their associated synovial sheaths pass through the carpal tunnel. The median nerve innervates five muscles in the hand: the first two lumbricals, the opponens pollicis, the abductor pollicis brevis and the flexor pollicis brevis. In MPS patients, CTS is generally caused by a combination of bone deformity, tenosynovial deposits, and GAG accumulation in the connective tissue of the flexor retinaculum, and is most common in MPS I, II, and VI [35, 39]. Typical signs are burning pain, tingling and numbness in the thumb, index and middle fingers, and the radial half of the ring finger [34, 38]. Cervical cord compression most frequently occurs in MPS I, IV, and VI and can be due to atlanto-axial instability, bony stenosis secondary to malformations of the spine and skull base, including odontoid dysplasia, or thickening of tissues surrounding the spinal cord [35, 40, 41]. Cervical cord compression in MPS patients may involve multiple levels, and can lead to compressive myelopathy, which can manifest as weakness, numbness, paresthesia, gait difficulty, and even paralysis or sudden death [39, 40].

Impaired sensation or pain and musculoskeletal abnormalities in the upper extremities may significantly affect ADL and self-care, and can lead to limitations in activity and social participation [7, 42]. CTS has been associated with increased pain and reduced physical functioning and overall QoL [43, 44], but evidence regarding the impact of CTS in MPS patients on PROs is limited [45].

Impairments in upper extremity function and pain in individuals with MPS can be evaluated using functional tests (e.g. goniometry, pinch and grip strength, 9-hole peg test), and/or PRO tools [8, 37, 46]. Diagnosing CTS can be difficult in MPS patients as it often progresses without typical symptoms, possibly due to masking symptoms, communication problems, insidious onset, and difficulties to perform nerve conduction studies due to the patients’ unusually small hands, often young age, and/or cognitive impairment [39, 47, 48]. Therefore, regular monitoring is important to identify CTS. Nerve ultrasound has been suggested as an alternative screening tool for CTS in these patients [47]. CTS can be treated successfully with surgical release of the median nerve, which should be performed before the median nerve is irreversibly damaged. Similarly, spinal cord compression should be surgically treated as recommended to prevent permanent damage to the spinal cord, and taking into account the considerable anesthetic risks in these patients [40, 41]. Pain can be managed with cognitive-behavioral strategies (e.g. relaxation training), physical strategies (e.g. exercise, physical activity), and pain medication [49–51].

### Challenges for assessing impairments in the senses in MPS

Although it is clear that MPS can have a considerable negative impact on each of the five senses, it remains unclear how patients perceive these impairments and how these affect their overall QoL. Literature on this subject is very sparse, and PRO tools for assessing impairments of the senses and their impact on QoL/ADL are generally not part of routine clinical care of MPS patients. A voting round during the MPS & the five senses meeting showed that only a minority of the attendees currently use these kinds of PRO tools in MPS patients in their practice. Nevertheless, PRO tools and physical tests (e.g. audiology tests, visual acuity tests, goniometry) can complement each other in decision-making for disease management. It is important to make a distinction between health and how patients perceive their health, which depends on how patients are coping with their impairments.

A better insight into how MPS affects the senses and how loss in one or more senses affects ADL and overall QoL in patients with this disease can be achieved by prospectively collecting data internationally in a standardized way, using a standard battery of tools. As interactions between the senses are important, all senses should be evaluated. It has been well established that loss in one of the senses can lead to compensatory plasticity and sharpening of other senses (e.g. enhanced auditory abilities and tactile perception in blind individuals [52, 53]). However, MPS patients with impairments in multiple senses may not be able to compensate. In patients with loss in one of the senses, it becomes more important to preserve functioning in the other senses. In addition to using sense-specific PRO tools, it is important to evaluate patients using a general QoL tool to assess overall QoL. This may reveal coping behaviors; i.e. when a specific sense QoL tool shows impairment, an overall QoL tool may present a score in the normal range.

At the MPS & the five senses meeting, there was general agreement that creating new or adapting existing PRO tools specifically for MPS patients is difficult due to the small patient number to test validity of these tools. Instead, existing PRO tools could be useful for evaluating these patients. However, selecting the most appropriate PRO tools for MPS patients is extremely challenging, because of the high number of available tools. The following criteria were perceived most important: 1) applicability to patients with MPS, 2) applicability to different countries (languages) and cultures, 3) ease of use (≤10 min to complete), 4) validation, and 5) availability of normative data.

With the above criteria in mind, the experts converged on the use of the five-level EuroQol five-dimensional questionnaire (EQ-5D-5 L) as the recommended general QoL tool to document changes in QoL in patients with MPS [54]. The EQ-5D-5 L is a simple and validated generic questionnaire that covers five dimensions of health: Mobility, Self-care, Usual activities, Pain/Discomfort and Anxiety/Depression. It is applicable to a wide range of health conditions, and has also been used in a number of studies involving patients with MPS [55–57]. Evaluation with a general well-established QoL measure, such as the EQ-5D-5 L, in combination with sense-specific tools will provide a better picture on the impact of impairments in the senses on QoL in MPS patients, and will better guide management.

### Selection of PRO tools for assessing the senses in MPS

After the meeting, a robust EMBASE literature search was performed in June 2018 to identify different PRO questionnaires used in other conditions related to the five senses, that may also be useful in the evaluation of sense impairments and their impact on QoL and/or ADL in MPS patients (Supplementary file 1). The searches were focused on tools for adult patients with no intellectual delay, who are able to complete questionnaires themselves.

The search strategy yielded a total of 421 unique hits, and identified a total of 33 tools for hearing, 30 for speech, 125 for vision, 49 for touch (including pain and upper limb function), and 15 for smell and taste. A selection of these PRO tools was made based on several criteria outlined in Supplementary file 1, including applicability/relevance for MPS, applicability in different countries (languages)/cultures, availability in English, ease of use, validation, and normative data. Table 1 provides an overview of the selected tools, including the most relevant criteria.

**Table 1:** Overview of PRO tools suitable for assessing impairment in the senses and overall health status in patients with MPS, with focus on adults and self-completion and based on the following criteria: applicability to patients with MPS, applicability to different countries and cultures, ease of use (≤10 min to complete), validated, and availability of normative data

Name tool	Abbreviation	Original target population	Time to complete	What does it measure?	Validation literature	Normative data literature	Used in MPS?	Language(s)	Target age
**Hearing & speech**									
**Hearing**									
Attitudes Toward Loss of Hearing Questionnaire	ALHQ	Hearing impairment, with or without hearing aids	±10 min	Attitudes toward hearing loss and hearing aids	Saunders G 2005 [58]	Saunders G 2005 [58]	No	English, Korean	Adults
Spatial Hearing Questionnaire	SHQ	Not disease-linked	±10 min	Perception of spatial hearing abilities/disabilities	Tyler RS 2009 [59]	Perreau AE 2014 [60]	No	English + 10 translations	NA
**Speech**									
Speech Handicap Index	SHI	Speech problems	5 min	Speech-related problems in daily life (psychosocial and speech function)	Rinkel RN 2008 [61]	Rinkel RN 2008 [61]	No	English, French, Dutch, Portuguese, Chinese	Adults
Voice Handicap Index	VHI	Voice disorders	5 min	Impact of voice disorders on QoL (functional, physical and emotional)	Francis DO 2017 [62]	Arffa RE 2012 [63]	No	English + 6 translations	Adults
Voice Outcome Survey	VOS	Uncompensated unilateral vocal fold paralysis	2–5 min	Vocal status and impact on daily activities	Gliklich RE 1999 [64]	Gliklich RE 1999 [64]		English, Chinese	Adults
**Vision**									
Visual Function Short Form	VF-8R	Cataract	5 min	Functional impairment caused by vision loss	Gothwal VK 2010 [65]	Gothwal VK 2010 (pre- vs post-op) [65]	No	English, Chinese	Adults
**Touch**									
**Upper limb function**									
Health Assessment Questionnaire	HAQ	Arthritis	5 min	Physical disability	Bruce B 2003 [66]	Bruce B 2003 [66]	Yes [8]^a^	English + 62 translations	Adults^d^
Quick Disabilities of the Arm, Shoulder and Hand Questionnaire	Quick-DASH	Upper-extremity disorders	2 min	Symptoms and ability to perform certain activities	Beaton DE 2005 [67]	Aasheim T 2014 [68]	No	50 languages	Adults
**Pain**									
Brief Pain Inventory Short Form	BPI-SF	Chronic or acute pain	5 min	Pain severity and impact of pain on daily functioning	Cleeland CS 2009 [69]	NA^c^	Yes [8]	English + 52 translations	Adults
West Haven - Yale Multidimensional Pain Inventory	WHYMPI	Chronic pain	5–10 min	Description of pain and how it affects the individual	Kerns RD 1985 [70]	https://www.va.gov/PAINMANAGEMENT/docs/	No	English + 9 translations	Adults
**Smell & taste**									
Chronic Sinusitis Survey^b^	CSS	Chronic sinusitis	5 min	Health status and treatment effectiveness in chronic rhinosinusitis	Gliklich RE 1995 [71]; Stavem K 2006 [72]	Gliklich RE 1997 [73]	No	English, Norwegian, Chinese, Turkish	Adults
**Health status**									
Five-level EuroQol five-dimensional questionnaire	EQ-5D-5 L	General population	< 5 min	Generic measure of health status for clinical and economic appraisal	Herdman M 2011 [54]	Szende A 2014 [74]	Yes [8]	> 120 languages	Adults

^a^An adapted version, the MPS-HAQ has been developed for patients with MPS [75]; ^b^One question of the CSS is not applicable to MPS, but specific for allergic rhinosinusitis

^c^The BPI-SF was included although no normative data are available, based on its ease of use and previous use in MPS

^d^A version of this questionnaire, i.e. the Child Health Assessment Questionnaire (CHAQ) is also available for children

*NA* Not available

## Conclusions

MPS can lead to considerable impairments in each of the five senses. However, current knowledge on the impact of sense impairments on QoL/ADL in patients with MPS remains very limited. Further research, i.e. collection of data in a standardized fashion using sense-specific PRO tools (e.g. those summarized in Table 1) and a general QoL tool such as the EQ-5D-5 L, is warranted and may provide a better insight in how and to what extent impairments in the senses affect ADL and the patients’ overall QoL. The current selection focuses on PRO tools for adults. However, as impairments in the senses are also prevalent in children and adolescents with MPS, it would be interesting to make a similar selection of tools that might be suitable for these populations. This would allow investigators to better follow up impairments in the senses in these patients over time and take appropriate actions.

## Supplementary information

**Additional file 1.** Details of literature search and selection of tools

## Data Availability

All data generated or analyzed during this study are included in this published article and its supplementary information files.

## Abbreviations

ADL: Activities of daily living
EQ-5D-5 L: Five-level EuroQol five-dimensional questionnaire
MPS: Mucopolysaccharidosis
PRO: Patient-reported outcomes
QoL: Quality of life
