# Supplementary material for: Assessing the impact of the five senses on quality of life in mucopolysaccharidoses
Source: Orphanet J Rare Dis. 2020 Apr 19;15:97. doi: 10.1186/s13023-020-01368-x (PMC7168888; doi:10.1186/s13023-020-01368-x)

**Supplementary file 1. Details of literature search and selection of tools**

1. **Literature search**

Literature searches in EMBASE were based on two different queries for each sense (all diseases), performed in June 2018

Search limits:

- Adults only
- All languages
- All years

*Queries for touch (including pain and upper limb function terms):*

- (('touch'/exp OR 'haptic perception' OR 'tactile perception' OR 'tactile performance' OR 'tactile sense' OR 'touch' OR 'touch perception' OR 'touch sense' OR 'touch impairment') AND ('patient-reported outcome'/exp OR 'patient reported outcome measures' OR 'patient-reported outcome' OR 'patient-reported treatment outcome' OR 'patientreported outcome' OR 'self-reported outcome' OR 'self-reported patient outcome' OR 'self-reported treatment outcome' OR 'selfreported outcome') AND (instrument OR 'tool'/exp OR scale OR measure OR 'validity'/exp OR 'validity' OR 'reliability'/exp OR 'psychometry'/exp OR 'psychometric screening' OR 'psychometric test' OR 'psychometrics' OR 'psychometry' OR 'psychomimetic technique')) AND ([adult]/lim OR [aged]/lim OR [middle aged]/lim)
- ('touch'/exp OR 'haptic perception' OR 'tactile perception' OR 'tactile performance' OR 'tactile sense' OR 'touch' OR 'touch perception' OR 'touch sense' OR 'pain and sensory' OR 'upper limb and sensory' OR 'upper limb movement and sensory' OR 'pain and touch' OR 'upper limb movement and touch') AND ('patient-reported outcome'/exp OR 'patient reported outcome measures' OR 'patient-reported outcome' OR 'patient-reported treatment outcome' OR 'patientreported outcome' OR 'self-reported outcome' OR 'self-reported patient outcome' OR 'self-reported treatment outcome' OR 'selfreported outcome') AND ('tool'/exp OR scale OR 'rating scale'/exp OR 'rating scale' OR 'measurement'/exp OR 'measurement' OR measure OR 'assessment'/exp OR 'battery'/exp OR instrument OR test OR rating OR 'score'/exp)

*Queries for smell and taste:*

- ('smelling and taste'/exp OR 'smelling and taste' OR 'smelling disorder'/exp OR 'olfaction disorder' OR 'olfaction disorders' OR 'smell disorder' OR 'smelling disorder' OR 'olfactory discrimination'/exp OR 'odor discrimination' OR 'odour discrimination' OR 'olfactory discrimination' OR 'smell acuity' OR 'smell discrimination' OR 'smelling sensitivity' OR 'taste disorder'/exp OR 'gustatory abnormality' OR 'gustatory disorder' OR 'gustatory distortion' OR 'gustatory disturbance' OR 'taste abnormality' OR 'taste anomaly' OR 'taste disorder' OR 'taste disorders' OR 'taste distortion' OR 'taste disturbance' OR 'taste'/exp OR 'gustation' OR 'gustatory perception' OR 'perception, gustatory' OR 'perception, taste' OR 'taste' OR 'taste perception' OR 'taste sense' OR 'tasting') AND ('patient-reported outcome'/exp OR 'patient reported outcome measures' OR 'patient-reported outcome' OR 'patient-reported treatment outcome' OR 'patientreported outcome' OR 'self-reported outcome' OR 'self-reported patient outcome' OR 'self-reported treatment outcome' OR 'selfreported outcome') AND (instrument OR 'tool'/exp OR scale OR measure OR 'validity'/exp OR 'validity' OR 'reliability'/exp OR 'psychometry'/exp OR 'psychometric screening' OR 'psychometric test' OR 'psychometrics' OR 'psychometry' OR 'psychomimetic technique')
- (('patient-reported outcome'/exp OR 'patient reported outcome measures' OR 'patient-reported outcome' OR 'patient-reported treatment outcome' OR 'patientreported outcome' OR 'self-reported outcome' OR 'self-reported patient outcome' OR 'self-reported treatment outcome' OR 'selfreported outcome') AND ('tool'/exp OR scale OR 'rating scale'/exp OR 'rating scale' OR 'measurement'/exp OR 'measurement' OR measure OR 'assessment'/exp OR 'battery'/exp OR instrument OR test OR rating OR 'score'/exp)) AND ('smelling and taste'/exp OR 'smelling and taste' OR 'smelling'/exp OR 'olfaction' OR 'olfactory acuity' OR 'olfactory analyzer' OR 'olfactory function' OR 'olfactory perception' OR 'olfactory reception' OR 'smelling' OR 'taste'/exp OR 'gustation' OR 'gustatory perception' OR 'perception, gustatory' OR 'perception, taste' OR 'taste' OR 'taste perception' OR 'taste sense' OR 'tasting' OR 'smelling disorder'/exp OR 'olfaction disorder' OR 'olfaction disorders' OR 'smell disorder' OR 'smelling disorder' OR 'taste disorder'/exp OR 'gustatory abnormality' OR 'gustatory disorder' OR 'gustatory distortion' OR 'gustatory disturbance' OR 'taste abnormality' OR 'taste anomaly' OR 'taste disorder' OR 'taste disorders' OR 'taste distortion' OR 'taste disturbance')

*Queries for vision:*

- (('visual disorder'/exp OR 'vision disorder' OR 'vision disorders' OR 'vision disturbance' OR 'visual disorder' OR 'visual disorders' OR 'visual disturbance' OR 'visual impairment'/exp OR 'impaired vision' OR 'sight impairment' OR 'vision defect' OR 'vision impairment' OR 'vision loss' OR 'vision, impaired' OR 'visual handicap' OR 'visual impairment' OR 'visual loss') AND ('patient-reported outcome'/exp OR 'patient reported outcome measures' OR 'patient-reported outcome' OR 'patient-reported treatment outcome' OR 'patientreported outcome' OR 'self-reported outcome' OR 'self-reported patient outcome' OR 'self-reported treatment outcome' OR 'selfreported outcome') AND (instrument OR 'tool'/exp OR scale OR measure OR 'validity'/exp OR 'validity' OR 'reliability'/exp OR 'psychometry'/exp OR 'psychometric screening' OR 'psychometric test' OR 'psychometrics' OR 'psychometry' OR 'psychomimetic technique')) AND ([adolescent]/lim OR [adult]/lim OR [aged]/lim OR [middle aged]/lim OR [very elderly]/lim OR [young adult]/lim)
- (('patient-reported outcome'/exp OR 'patient reported outcome measures' OR 'patient-reported outcome' OR 'patient-reported treatment outcome' OR 'patientreported outcome' OR 'self-reported outcome' OR 'self-reported patient outcome' OR 'self-reported treatment outcome' OR 'selfreported outcome') AND ('tool'/exp OR scale OR 'rating scale'/exp OR 'rating scale' OR 'measurement'/exp OR 'measurement' OR measure OR 'assessment'/exp OR 'battery'/exp OR instrument OR test OR rating OR 'score'/exp)) AND ('vision'/exp OR 'capacity, visual' OR 'central vision' OR 'figural aftereffect' OR 'half vision' OR 'ocular vision' OR 'optic perception' OR 'perception, optic' OR 'perception, visual' OR 'perceptual closure' OR 'phosphene' OR 'phosphenes' OR 'twilight vision' OR 'vision' OR 'vision, entoptic' OR 'vision, ocular' OR 'visual capacity' OR 'visual detection' OR 'visual function' OR 'visual perception' OR 'visual performance' OR 'visual process' OR 'visual sensation' OR 'visual disorder'/exp OR 'vision disorder' OR 'vision disorders' OR 'vision disturbance' OR 'visual disorder' OR 'visual disorders' OR 'visual disturbance' OR 'vision and sensory') AND [humans]/lim

*Queries for speech*

- (('speech disorder'/exp OR 'logopathy' OR 'speech defect' OR 'speech deficiency' OR 'speech disorder' OR 'speech disorders' OR 'speech disturbance' OR 'speech impairment' OR 'speech impediment' OR 'speech problem' OR 'speech-language pathology') AND ('patient-reported outcome'/exp OR 'patient reported outcome measures' OR 'patient-reported outcome' OR 'patient-reported treatment outcome' OR 'patientreported outcome' OR 'self-reported outcome' OR 'self-reported patient outcome' OR 'self-reported treatment outcome' OR 'selfreported outcome')) AND ([adult]/lim OR [aged]/lim OR [middle aged]/lim OR [very elderly]/lim OR [young adult]/lim)
- (('patient-reported outcome'/exp OR 'patient reported outcome measures' OR 'patient-reported outcome' OR 'patient-reported treatment outcome' OR 'patientreported outcome' OR 'self-reported outcome' OR 'self-reported patient outcome' OR 'self-reported treatment outcome' OR 'selfreported outcome') AND ('tool'/exp OR scale OR 'rating scale'/exp OR 'rating scale' OR 'measurement'/exp OR 'measurement' OR measure OR 'assessment'/exp OR 'battery'/exp OR instrument OR test OR rating OR 'score'/exp)) AND ('speech disorder'/exp OR 'speech defect' OR 'speech deficiency' OR 'speech disorder' OR 'speech disorders' OR 'speech disturbance' OR 'speech impairment' OR 'speech impediment' OR 'speech problem' OR 'speech-language pathology' OR 'logopathy')

*Queries for hearing*

- (('hearing disorder'/exp OR 'hearing impairment'/exp OR 'auditory defect' OR 'bilateral deafness' OR 'deaf' OR 'deafness' OR 'deafness, bilateral' OR 'hearing damage' OR 'hearing defect' OR 'hearing difficulty' OR 'hearing impairment' OR 'hearing loss' OR 'hearing loss, bilateral' OR 'hearing loss, central' OR 'hearing loss, high frequency' OR 'hypacusia' OR 'hypacusis' OR 'hypoacousia' OR 'hypoacusis' OR 'impaired hearing') AND ('patient-reported outcome'/exp OR 'patient reported outcome measures' OR 'patient-reported outcome' OR 'patient-reported treatment outcome' OR 'patientreported outcome' OR 'self-reported outcome' OR 'self-reported patient outcome' OR 'self-reported treatment outcome' OR 'selfreported outcome') AND (instrument OR 'tool'/exp OR scale OR measure OR 'validity'/exp OR 'validity' OR 'reliability'/exp OR 'psychometry'/exp OR 'psychometric screening' OR 'psychometric test' OR 'psychometrics' OR 'psychometry' OR 'psychomimetic technique')) AND ([adolescent]/lim OR [adult]/lim OR [aged]/lim OR [middle aged]/lim OR [very elderly]/lim OR [young adult]/lim)
- (('patient-reported outcome'/exp OR 'patient reported outcome measures' OR 'patient-reported outcome' OR 'patient-reported treatment outcome' OR 'patientreported outcome' OR 'self-reported outcome' OR 'self-reported patient outcome' OR 'self-reported treatment outcome' OR 'selfreported outcome') AND ('tool'/exp OR scale OR 'rating scale'/exp OR 'rating scale' OR 'measurement'/exp OR 'measurement' OR measure OR 'assessment'/exp OR 'battery'/exp OR instrument OR test OR rating OR 'score'/exp)) AND ('hearing disorder'/exp OR 'hearing disorder' OR 'hearing disorders' OR 'hearing impairment'/exp OR 'auditory defect' OR 'bilateral deafness' OR 'deaf' OR 'deafness' OR 'deafness, bilateral' OR 'hearing damage' OR 'hearing defect' OR 'hearing difficulty' OR 'hearing impairment' OR 'hearing loss' OR 'hearing loss, bilateral' OR 'hearing loss, central' OR 'hearing loss, high frequency' OR 'hypacusia' OR 'hypacusis' OR 'hypoacousia' OR 'hypoacusis' OR 'impaired hearing' OR 'hearing and sensory')

1. **Selection process of PRO tools for the senses identified by literature searches**


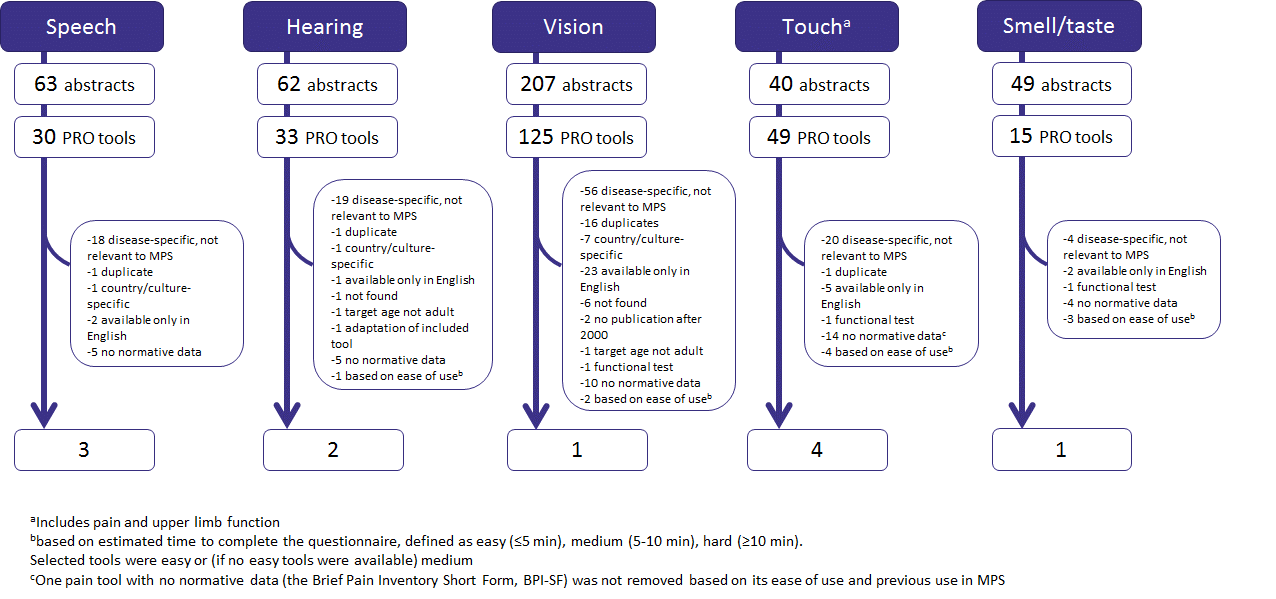

Supplement: Supplementary file 1 — Additional file 1. Details of literature search and selection of tools [file 13023_2020_1368_MOESM1_ESM.docx]
